# Esmolol response in septic shock patients in relation to vascular waterfall phenomenon measured by critical closure pressure and mean systemic filling pressure: a prospective observational study

**DOI:** 10.1186/s40560-021-00587-z

**Published:** 2022-01-04

**Authors:** Zehan Liu, Chuanliang Pan, Jianping Liu, Hui Liu, Hui Xie

**Affiliations:** 1 Department of Surgical Intensive Care Unit, The Third People’s Hospital of Chengdu, Affiliated Hospital of Southwest Jiaotong University, Chengdu, China; 2 Section for HepatoPancreatoBiliary Surgery, Department of General Surgery, The Third People’s Hospital of Chengdu, Affiliated Hospital of Southwest Jiaotong University, Chengdu, China

**Keywords:** Septic shock, Esmolol, Critical closure pressure, Mean systemic circulation filling pressure, Vascular waterfall

## Abstract

**Background:**

Bedside measurements of critical closure pressure (Pcc) and mean systemic circulation filling pressure (Pmsf) were utilized to evaluate the response to esmolol in septic shock patients, in relation to the vascular waterfall phenomenon and body oxygen supply and demand.

**Methods:**

This prospective observational self-controlled study included patients with septic shock, newly admitted to the intensive care unit, between August 2019 and January 2021. Pcc and Pmsf, along with the heart rate and other hemodynamic indicators were observed and compared before and 1 h after esmolol IV infusion.

**Results:**

After 24 h of initial hemodynamic optimization, 56 patients were finally enrolled. After start of esmolol infusion, patients had a significant decrease in cardiac index (CI) (4.0 vs. 3.3 L/min/m^2^, *P* < 0.001), a significant increase in stroke index (SI) (34.1 vs. 36.6 mL/m^2^, *P* < 0.01), and a significant decrease in heart rate (HR) (116.8 vs. 90.6 beats/min, *P* < 0.001). After 1 h of treatment with esmolol, patients had a significant increase in Pcc (31.4 vs. 36.7 mmHg, *P* < 0.01). The difference between Pcc and Pmsf before and after treatment was statistically different (4.0 vs. 10.0 mmHg, *P* < 0.01). After heart rate control with esmolol, the patients had a significant increase in the body circulation vascular resistance indices (RIs) (15.14 vs. 18.25 mmHg/min/m^2^/L, *P* < 0.001). There was an increase in ScvO2 in patients after treatment with esmolol, but the difference was not statistically significant (68.4% vs. 69.8%, *P* > 0.05), while Pcv-aCO2 was significantly lower (6.3 vs. 4.9 mmHg, *P* < 0.001) and patients had a significant decrease in blood lactate levels (4.0 vs. 3.6 mmol/L, *P* < 0.05).

**Conclusion:**

Patients with septic shock whose heart rate is greater than 95 beats/min after hemodynamic optimization were treated with esmolol, which could effectively control heart rate and reduce CI, as well as improve Pcc and increase the difference between Pcc and Pmsf (known as “vascular waterfall” phenomenon), without affecting MAP, CVP, Pmsf and arteriovenous vascular resistance, and improve the balance of oxygen supply and demand in the body.

## Background

The early clinical management of patients with septic shock depends on the hemodynamic management and optimization. The pathophysiological characteristics of septic shock include a waterfall-like inflammatory response, which is often accompanied by sympathetic hyperactivation, continuously elevated catecholamine levels, myocardial depression, vascular hyporesponsiveness and autonomic nerve dysfunction [1–3]. In addition, the continuous increase of catecholamine levels will cause myocardial ischemia, calcium overload, oxidative stress, metabolic disorders, mitochondrial dysfunction, cell dysfunction or death, thereby endangering the patient in many ways [3].

The phenomenon of "vascular waterfall", hemodynamic state of collapsible vessels, different from classic Poiseuille law and similar to waterfalls in nature, has been studied since the 1960s [4–7]. In contrast with traditional flow, dependant on cardiac output and systemic vascular resistance, flow of the waterfall depends on the pressure difference between the upstream source (arterial blood pressure, ABP) and the top of the waterfall (critical closure pressure, Pcc), while the pressure at the bottom of the waterfall (mean systemic filling pressure, Pmsf) and the downstream resistance do not affect the flow [8]. Under these circumstances, the whole vasculature would be divided into arterial units, microcirculation units and venous units, as opposed to the classic hemodynamics that compares the entire vascular system to a set of stiff ducts, which no longer seems suitable [9]. Clinically, the treatment of septic shock often encounters phenomena that are difficult to explain by classic circulation hemodynamic theory. For example, under the premise of ensuring average arterial pressure and cardiac displacement, many patients will still develop acute kidney injury or acute renal failure, and oliguria or anuria may occur [2, 10].

Based on that, blocking the side effects caused by high catecholamine levels helps to improve the prognosis of patients with septic shock [11, 12]. Esmolol is a highly selected, ultra short acting β1-receptor blocker that blocks the high metabolism caused by high catecholamines, reduces oxygen consumption, protects the myocardium, and modulates the body's inflammatory response, having already demonstrated the potential in the treatment of septic shock [13]. Currently, esmolol has been reported and recommended in many studies for the treatment of heart rate control after fluid resuscitation, with the ability to significantly improve hemodynamic status and prognosis. However, these studies do not seem to fully explain why the systemic resistance of patients is significantly improved (increased) with esmolol and the hemodynamic mechanisms underlying the patients' improved microcirculatory perfusion and renal function remain unclear [14]. These difficulties provide rationale to further study the changes of renal microcirculation, and monitoring P_CC_ and P_MSF_ is a good breakthrough opportunity to connect the macrocirculation and microcirculation.

Consequently, by explaining the pressure gradient between arteriolar closure pressure Pcc and the mean venous filling pressure Pmsf at the level of small veins, vascular waterfall phenomenon is an important discovery in the study of the microcirculation system, and with the further research of many scholars this phenomenon can be measured stably and reliably. In recent years, more and more studies have been conducted to explore and explain related circulation problems by measuring vascular waterfall, which is an important bridge between the study of macrocirculation and microcirculation [15]. Therefore, in this paper, the hemodynamic response to esmolol in septic shock patients was studied utilizing bedside measurement of Pcc and Pmsf. We hypothesized that because esmolol does not affect systemic arteriovenous vascular resistance it can help to safely restore the body's vascular waterfall phenomenon.

## Materials and methods

### Study design, settings and patients

The study, which adopts a prospective observational method, enrolled patients with septic shock, newly admitted to the intensive care unit (ICU) of the Department of Critical Care Medicine, Chengdu Third People's Hospital, between August 2019 and January 2021. Patients diagnosed with septic shock according to the Sepsis-3 criteria [1] underwent endotracheal intubation, invasive ventilator assisted ventilation, pulse indicator continuous cardiac discharge monitoring (PICCO) catheter and deep vein catheter according to the needs of their condition. Inclusion criteria were age ≥ 18 years, 24 h after ICU admission, heart rate greater than 95 bpm after appropriate hemodynamic therapy, need for norepinephrine (≥ 0.10 μg/kg/min) to maintain mean arterial pressure (MAP > 65 mmHg), Global End-Diastolic Volume Index (GEDVI) > 700 mL/m^2^, and Intrathoracic Blood Volume Index (ITBVI > 850 mL/m^2^). Diagnostic process of septic shock included the following: for patients with infection or suspected infection, if their sequential organ failure assessment (SOFA) score was ≥ 2 or doctors suspected sepsis, we further evaluated whether there is an evidence of organ dysfunction; if the patient's SOFA score was ≥ 2 or the new score was ≥ 2, it was diagnosed as sepsis. Septic shock was diagnosed if patients with sepsis still needed to maintain blood pressure MAP ≥ 65mmhg or blood lactate levels > 2 mmol/L after sufficient fluid resuscitation.

Exclusion criteria were previous treatment with β blockers before or within 24 h of ICU admission, severe valvular disease, congenital heart disease or cardiomyopathy, severe pulmonary bullae or spontaneous pneumothorax, need for inotropic agents or severe cardiac dysfunction (CI < 2.2 L/min/m^2^ and GEDVI > 700 mL/m^2^ and ITBVI > 850 mL/m^2^), adequate sedation and analgesia for less than 36 h, length of stay in the ICU less than 48 h, surgery or re-operation within 48 h after admission to ICU, and pregnancy.

Study was approved by the medical ethics review committee of The Third People's Hospital of Chengdu, Affiliated Hospital of Southwest Jiaotong University (Approval No. [2019] S-22), and informed consent was obtained from patients or their next of kin.

### Patient management and measurement of hemodynamics

Within 24 h from admission to the ICU, the patients received the primary treatment according to the following scheme: (1) Hemodynamic therapy with shock resuscitation immediately after the patient was admitted to the ICU. Hemodynamic treatment objectives were: CI > 3 L/min/m^2^ and GEDVI > 700 mL/m^2^, ITBVI > 850 mL/m^2^; MAP > 65 mmHg; central venous oxygen saturation (S_cv_O_2_) ≥ 70%; and urine output > 0.5 mL/kg/h. (2) Respiratory support therapy: Mechanical ventilation was performed in volume control mode (AVEA, CareFusion, California, US) with a target tidal volume of 6 to 8 ml/kg or less. (3) Sedation and analgesia: Dexmedetomidine or midazolam was used for continuous intravenous infusion sedation, fentanyl or butorphanol was used for analgesia; (4) Others: Rational use of antibiotics to fight infection, insulin control of blood sugar, dynamic monitoring of blood lactic acid level, maintaining acid–base electrolyte balance, etc.

24 h after admission to ICU, patients whose heart rate was more than 95 beats/min after hemodynamic optimization and needed norepinephrine (≥ 0.10 μg/kg/min) to maintain blood pressure began to receive intravenous esmolol (Esmolol Hydrochloride Injection; Qilu Pharmaceutical, Jinan, China) to control heart rate. The loading dose was 0.25–0.5 mg/kg (intravenous injection, administration time at least 1 min) and the maintenance dose was 0.05 mg/kg/min, IV (intravenous pumping), which was dynamically adjusted by the doctor in charge according to the heart rate and the changes of the disease. 24 h after the patient enters the ICU, the target heart rate was controlled at 80–94 bpm until he leaves the ICU [14].

Through continuous sedation at the bedside until the patient until no spontaneous breathing for 12 s, steady-state CO, CVP and MAP were measured over the last 3-s of 12-s inspiratory hold maneuvers at plateau pressures of 5, 15, 25 and 35 cmH_2_O. The ventricular output (VO) curve and venous return (VR) curve were constructed for the 4 pairs of CO, MAP values and CO, CVP values obtained from the 4 plateau pressures. A linear regression line was fitted through these data points. When the flow velocity was zero, the cutoff values of the pressure axis were Pcc and Pmsf, respectively [16, 17].

CVP, MAP, HR, GEDVI, ITBVI, extravascular lung water (EVLWI), cardiac stroke volume index (SI), CI, ScvO2, Pcc, Pmsf, VO curve slope, VR curve slope, blood lactate level (Lac), central venous-to-arterail carbon dioxide difference (Pcv-aCO_2_), dosage of norepinephrine and urine output per hour were observed and recorded before and 1 h after esmolol treatment (that is, 24 and 25 h after ICU admission). During the observation period, the patient was no longer treated with fluid resuscitation, and only the necessary drugs were given to maintain infusion. The systemic vascular resistance index (RIs) was defined as RIs = (MAP-CVP)/CI; arterial vascular resistance index (RIa) = (MAP-Pcc)/CI; venous vascular resistance index (RIv) = (Pmsf-CVP)/CI. Vascular waterfall was confirmed in cases of Pcc > Pmsf.

### Statistical analysis

Statistical analyses were performed using SPSS 22.0 (IBM, Armonk, NY, US). The measurement data are presented as mean ± SD (*x* ± *s*). The data before and after treatment with esmolol were compared by a paired-sample *t* test. The least square method was used to fit the linear regression of Pcc and Pmsf. Differences with a *P* value of less than 0.05 were considered statistically significant.

## Results

### Patient's basic information

**Fig. 1:**
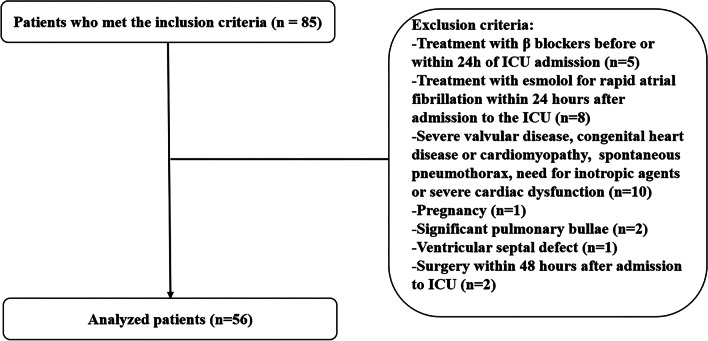
Patient flowchart

The inclusion–exclusion flow diagram of the study enrollment is demonstrated on Fig. 1. Fifty-six patients were finally enrolled, including 40 males and 16 females; their ages ranged from 29 to 88 years, with a mean of (62.6 ± 13.7) years; 28 of them had pulmonary infections, 23 had abdominal infections, and 5 had pyelonephritis. The sequential organ failure assessment scores (SOFA) score was 12.1 ± 3.6 at ICU admission and 14.8 ± 2.5 after 24 h in ICU. The 48-h mortality rate after entering the ICU was zero and no esmolol-related bradycardia (< 50 times/min) occurred.

### Haemodynamic response to esmolol in septic shock patients

**Fig. 2:**
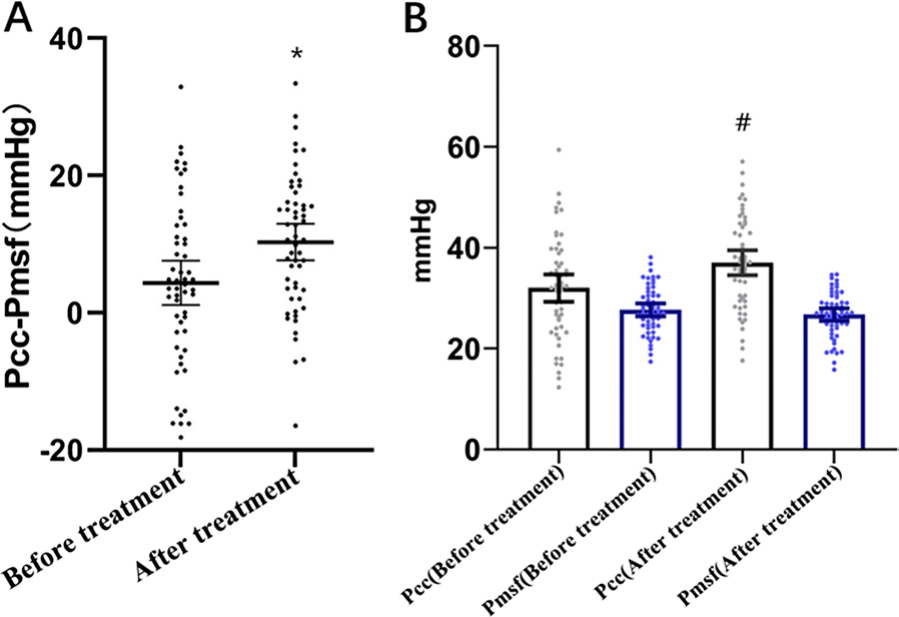
**a** Comparation of difference of PCC and Pmsf before and after esmolol treatment (*P = 0.009); **b** Comparation of PCC and Pmsf before and after esmolol treatment (Pcc: ^#^*P* = 0.008). The bold lines and bars represent means, the error bars represent 95% confidence intervals

A total of 85 patients satisfied inclusion criteria, of them 56 enrolled patients were treated with appropriate hemodynamic therapy, had a heart rate > 95 bpm 24 h after admission to the ICU, required norepinephrine (≥ 0.10 μg/kg/min) to maintain MAP > 65 mmHg, and had a GEDVI > 700 mL/m^2^ and ITBVI > 850 mL/m^2^. At this time, we immediately administered esmolol to control the heart rate and controlled the patient's target heart rate to 80–94 bpm. We measured hemodynamic parameters before esmolol treatment and after esmolol treatment for 1 h (Table 1, Fig. 2).

**Table 1:** Comparison of hemodynamic data before and after esmolol administration in patients with septic shock

Variables	Before treatment		After treatment		*P*
	Mean	SD	Mean	SD	
CI (L/min/m^2^)	4.0	0.5	3.3	0.5	0.000
SI (mL/m^2^)	34.1	4.9	36.6	5.6	0.008
HR (beats/min)	116.8	10.1	90.6	4.8	0.000
MAP (mmHg)	71.4	3.1	72.0	1.9	0.309
CVP (mmHg)	12.4	1.5	12.8	1.7	0.273
GEDVI (mL/m^2^)	748.0	25.7	751.9	25.8	0.426
ITBVI (mL/m^2^)	906.1	47.0	903.0	41.6	0.676
EVLWI (mL/kg)	13.3	4.7	13.4	3.9	0.888
Pcc (mmHg)	31.4	10.9	36.7	9.4	0.008
Pmsf (mmHg)	27.7	4.7	26.7	4.5	0.293
Slope of VO curve (L/min/mmHg)	0.109	0.040	0.104	0.044	0.511
Slope of VR curve (L/min/mmHg)	-0.294	0.136	-0.290	0.210	0.906
Pcc-Pmsf (mmHg)	4.0	12.1	10.0	10.0	0.009
Pmsf-CVP (mmHg)	15.3	4.8	13.9	5.0	0.179
RIs (mmHg·min·m^2^/L)	15.14	2.02	18.25	2.79	0.000
RIa (mmHg·min·m^2^/L^1^)	10.14	2.98	10.80	3.04	0.264
RIv (mmHg·min·m^2^·L^−1^)	3.91	1.30	4.25	1.57	0.231
RIa + RIv (mmHg·min·m^2^/L^1^)	14.05	3.51	15.05	3.40	0.145
Norepinephrine dosage (μg/kg/min)	0.20	0.05	0.20	0.04	0.444
Urine output per hour (mL/kg/h)	1.40	0.49	1.45	0.54	0.589

**Fig. 3:**
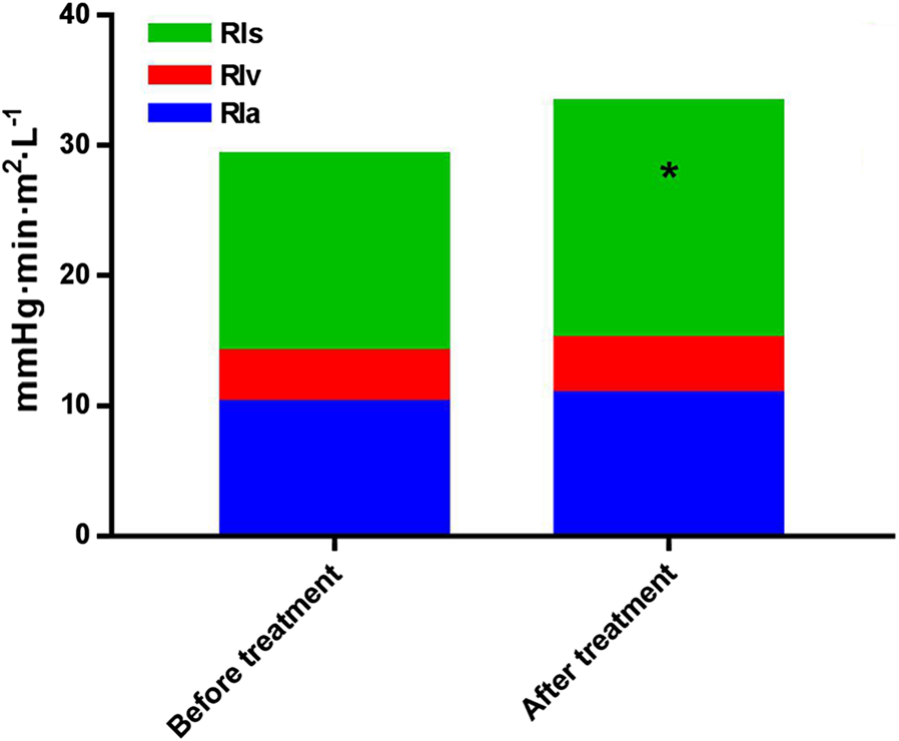
Changes of vascular resistance index

According to vascular waterfall theory, we monitored the changes of vascular resistance index (RI) in patients before and after esmolol treatment. Although after esmolol treatment systemic RI was significantly higher than before treatment (18.25 vs. 15.14 mmHg·min/m^2^/L, *P* < 0.001), arterial RIa (10.14 vs. 10.80, *P* = 0.264), venous RIv (3.91 vs. 4.25, *P* = 0.231) and RIa + RIv (14.05 vs. 15.05, *P* = 0.145) did not change significantly compared to before treatment (Fig. 3).

After heart rate control with esmolol, patient CI decreased significantly (4.0 vs. 3.3 L/min/m2, *P* < 0.001), while SI, Pcc and Pcc-Pmsf were significantly increased (34.1 vs. 36.6 mL/m^2^, *P* = 0.008; 31.4 vs. 36.7, *P* = 0.008; 4.0 vs. 10.0, *P* = 0.009). The decrease in CI was mainly due to a decrease in heart rate after esmolol treatment (116.8 vs. 90.6 bpm, *P* < 0.001). Esmolol did not affect the levels of MAP (71.4 vs. 72.0 mmHg, *P* = 0.309) and CVP (12.4 vs. 12.8 mmHg, *P* = 0.273). Simultaneously, before and after esmolol treatment, patients' PiCCO volume indexes GEDVI (748.0 vs. 751.9 mL/m^2^, *P* = 0.426), ITBVI (906.1 vs. 903.0 mL/m^2^, *P* = 0.676) and EVLWI (13.3 vs. 13.4 mL/kg, *P* = 0.008) were not changed either.

We also monitored the oxygen supply and demand balance indicators before and after esmolol administration in 56 patients admitted to the hospital, and the results are shown in Table 2. After treatment with esmolol, patients had an increase in ScvO_2_, but the difference was not statistically significant (68.4% vs. 69.8%, *P* = 0.063), while Pcv-aCO_2_ was significantly decreased (6.3 vs. 4.9 mmHg, *P* < 0.001) and patients had a significant decrease in blood lactate levels (4.0 vs. 3.6 mmol/L, *P* = 0.012), suggesting that esmolol improves the tissue oxygen metabolism of the body. Combined with the changes in hemodynamic indicators, the difference between Pcc and Pmsf was significantly increased, and it seems that the improvement in oxygen metabolism was associated with the restoration of vascular waterfalls and improved microcirculatory perfusion in more tissues and organs.

**Table 2:** Comparison of oxygen supply and demand balance data before and after esmolol administration in patients with septic shock

Variables	Before treatment		After treatment		*P*
	Mean	SD	Mean	SD	
S_cv_O_2_ (%)	68.4	4.7	69.8	3.2	0.063
P_cv-a_CO_2_ (mmHg)	6.3	1.7	4.9	1.6	0.000
Lac (mmol/L)	4.0	1.0	3.6	0.9	0.012

### Correlation analysis of Pcc-Pmsf and lactate levels

**Fig. 4:**
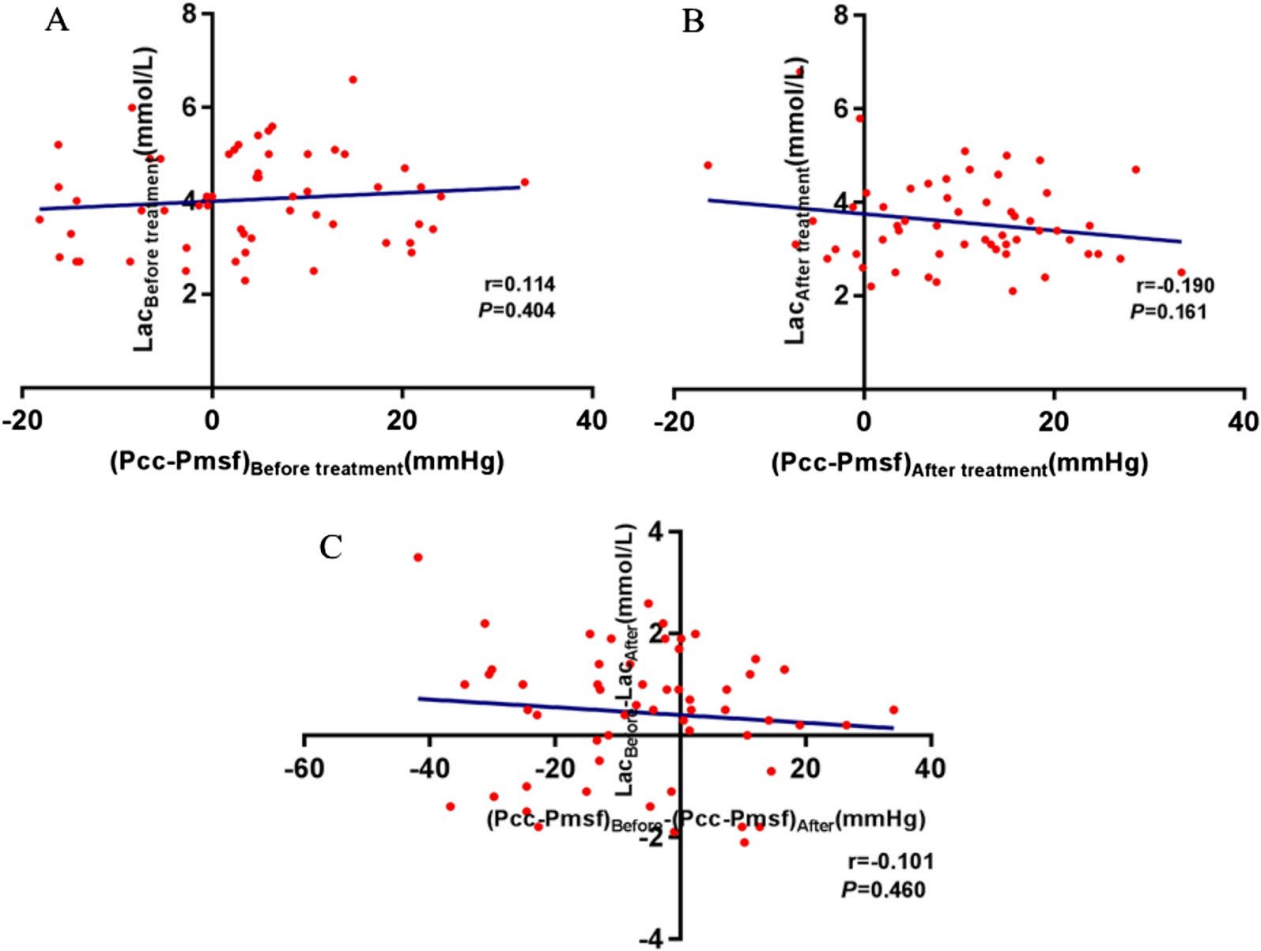
Correlation analysis of Pcc-Pmsf and lactate levels. **a** Before esmolol treatment (*P* = 0.404); **b** After esmolol treatment (*P* = 0.161); **c** The relationship between the changes of vascular waterfall and the changes of lactic acid before and after treatment (*P* = 0.460). The blue line is linear regression

After administration of estomol, patients had a significant decrease in blood lactate levels (4.0 vs. 3.6 mmol/L, *P* = 0.012). However, there was no significant correlation found directly between the Pcc-Pmsf and lactate levels, before or after treatment (Fig. 4).

## Discussion

This study utilized bedside measurements of critical closure pressure (Pcc) and mean systemic circulation filling pressure (Pmsf) to evaluate the response to esmolol in septic shock patients, in relation to the vascular waterfall phenomenon and body oxygen supply and demand. It was found that patients with septic shock whose heart rate is greater than 95 beats/min, benefited from addition of esmolol after hemodynamic optimization, demonstrating a significant decrease in CI and a HR. The difference between Pcc and Pmsf before and after treatment was statistically significant, indicating a marked increase in the body circulation vascular resistance indices and successful restoration of the “Vascular Waterfall”.

According to the classic Poiseuille law, arterial blood pressure is determined by the cardiac output and systemic vascular resistance, that is, the flow is proportional to the pressure gradient of the pipeline. However, an increasing number of studies have reported the presence of vascular waterfalls in vascular beds [4–7]. When there is a vascular waterfall phenomenon, Poiseuille's law cannot be applied to the entire vascular beds. Just like a waterfall in nature, the flow of a waterfall has nothing to do with the pressure difference between the top and bottom of the waterfall and the resistance downstream. It only depends on the pressure difference between the upstream of the waterfall and the top of the waterfall and the flow resistance of the upstream of the waterfall and there is no resistance from the top of the waterfall to the bottom of the waterfall [9]. In the vascular waterfall, the pressure at the top of the waterfall is Pcc and the pressure at the bottom is Pmsf. Some studies suggest that Pcc may act in the anterior arterioles of capillaries and Pmsf acts in the posterior venules of capillaries [18, 19]. Because the vascular waterfall is mainly located in the microcirculation, it can reflect the tissue perfusion. Therefore, if the vascular waterfall disappears, the tissue perfusion disappears. In our study we hypothesized that because esmolol does not affect systemic arteriovenous vascular resistance it can help to safely restore the body's vascular waterfall phenomenon. Monitoring during the study showed that although systemic vascular resistance (RIs) was significantly increased after esmolol treatment, vascular resistance (RIa + RIv) did not change, thus confirming our initial hypothesis.

Based on understanding of waterfall phenomenon, monitoring of Pcc and Pmsf, which can be performed at the bedside in the ICU, gives the opportunity for hemodynamic management to shift from macrocirculation to microcirculation. Draw VO curve and VR curve, respectively, through cardiopulmonary interaction, end-inspiratory breath-holding and continuous increase of end-inspiratory airway platform pressure, and linear fitting can be used to obtain the corresponding Pcc and Pmsf when CO is zero [16, 17]. This method has been confirmed by numerous studies, and it is an accurate, reliable, repeatable, non-invasive and convenient method for measurement. However, different organs or tissues of the body have different Pcc. The perfusion pressure of tissues and organs depends on the difference between MAP and its corresponding Pcc, which determines the different distribution of blood flow in the body and ensures the perfusion of important organs. The Pcc measured in this study reflects the average Pcc of the body's tissues and organs [9].

So far, many evidence-based medical sources, including Chinese guidelines for patients with septic shock, recommend esmolol to control the increased heart rate of septic shock patients after hemodynamic optimization, which can improve the hemodynamic status, microcirculation perfusion, renal function and prognosis of patients [10, 14]. However, these studies mainly focus on the macrocirculation level and the mechanism of esmolol to improve the microcirculation and tissue perfusion is still unclear. From already published research data [10, 14], it seems that esmolol can significantly increase systemic vascular resistance (CI decreased, MAP unchanged). These are phenomena that are difficult to explain in clinical work. In 2017, an animal study conducted by Liu Dawei's team at Peking Union Medical College Hospital in China found that esmolol not only restored the vascular waterfall that had disappeared in the kidneys during septic shock, but also improved the prognosis [20]. Therefore, in this study, we tried to further investigate the response to esmolol in relation to the increase of heart rate in septic shock patients by bedside measurement of Pcc and Pmsf, focusing on how to improve tissue perfusion and prognosis, and how to influence the changes of vascular resistance.

In this study, we found that after treatment with esmolol, as heart rate was effectively controlled, patients showed a significant decrease in CI and no statistical changes in MAP. However, due to the existence of the vascular waterfall, the patient's arterial and venous resistance did not change. This finding seems to explain the problem of previous studies that the systemic vascular resistance increased significantly after the use of esmolol [10, 14]. The study also found that Pcc levels were significantly elevated after the use of esmolol and the difference between Pcc and Pmsf increased. Since the Pcc of this study was the average Pcc of the whole body, it means that more tissues or organs of the body restored the vascular waterfall. This has also been further confirmed in the monitoring of oxygen supply–demand balance, that is, esmolol improved the body's state of oxygen supply–demand balance. MAP is a key factor for tissue organ perfusion and CI is a prerequisite. In previous reports, MAP remained unchanged and CI decreased after esmolol administration in septic shock patients, whereas tissue perfusion (hypoglossal circulation and renal function) was significantly improved [10, 14]. This is a phenomenon that these studies are difficult to explain. In addition, these findings in the present study seem to precisely explain the phenomenon. The kidney is the first organ involved when shock occurs and the last organ that recovers when shock is corrected [20]. This seems to explain why the hourly urine output of patients did not increase significantly after esmolol use in this study.

It is interesting to note, that the waterfall (Pcc-Pmsf) could be negative in some cases, as demonstrated in Fig. 1a. First, from the point of methodology, PCC in this study is the average PCC of tissues and organs in the whole body, not the PCC of an organ. Pcc-Pmsf is within a certain range, so the positive value of the difference increases, which seems to indicate that more tissues or organs have restored microcirculation perfusion, while Pcc-Pmsf itself cannot reflect the microcirculation perfusion of an organ or tissue. Therefore, Pcc-Pmsf reflects a trend of systemic microcirculation perfusion—in other words, can mainly reflect absence or presence of changes in microcirculation perfusion, but cannot reflect the exact degree of changes, as degree of changes in microcirculation perfusion also depends on perfusion pressure (MAP-PCC) and cardiac displacement (CO). Second, the fact that negative value of Pcc-Pmsf can indicate the poor microcirculation and blood perfusion in tissues and organs of the whole body is also reflected in our further data analysis; that is, in 36 cases before treatment Pcc-Pmsf was positive, and after esmolol treatment in 46 cases Pcc-Pmsf was positive (number of cases with negative Pcc-Pmsf decreased). It can also be clearly seen from Fig. 2a that after esmolol treatment, more patients showed the change of PCC value greater than PMSF, so Pcc-Pmsf coefficient was changed. Finally, in the study published by Liu Dawei's team in 2017 [20], it was reported that the mean values of baseline renal PCC in the control group and esmolol group were negative (respectively, − 6.7 ± 10.1 vs. − 2.5 ± 16.0), and the mean value of PMSF was positive (25.8 ± 2.2 vs. 27.1 ± 4.2), suggesting that the phenomenon of renal vascular waterfall disappeared due to septic shock. It seems that it can also explain what kind of state the patient is in when Pcc-Pmsf is negative.

Unfortunately, we did not observe any association between Pcc-Pmsf and Lac before and after esmolol treatment. The reason may be the restoration of vascular waterfall phenomenon, as with the difference of Pcc-Pmsf from negative to positive, tissue perfusion also changed, and microcirculation perfusion improved. However, with the increase of Pcc, the difference of vascular waterfall Pcc-Pmsf further increased (Pmsf did not change significantly in this study), and the tissue perfusion pressure (MAP-PCC) decreased significantly (no significant change occurred before and after MAP). In this study, CI also decreased significantly after esmolol treatment, so tissue perfusion level decreased instead. Therefore, no link to the changes in Lac was found during the given timeframe.

This study still has certain limitations: First, this was a nonrandomized controlled study; because clinical guidelines for sepsis in China and evidence-based data is currently available to recommend esmolol for patients with septic shock whose heart rate remains greater than 95 bpm after hemodynamic optimization [14, 21, 22], it would be unfavorable and not ethical to perform randomized controlled studies in these patients again. Therefore, this study adopted a self-controlled research method to allow enrolled patients all to receive timely treatments recommended by evidence-based medicine. Second, this study only compared hemodynamic, oxygen supply–demand balance data obtained 1 h after the use of esmolol and it was not a continuous dynamic evaluation. No comparison was made between the use of esmolol for 6 h, 12 h, 24 h or even longer. The main reasons for this are as follows: (1) intravenous esmolol has a rapid onset of action within 1 min after initial infusion. Therefore, the data after using esmolol for 1 h can accurately reflect the treatment effect and there was no need to wait longer; (2) measurements of Pcc and Pmsf required the patients to be under sedation, free of spontaneous breathing, invasive ventilator assisted ventilation and able to hold his/her breath for 12 s. Thus, measuring data for a longer period of time will cause a lot of unnecessary sedation and prolong the mechanical ventilation time, which were disadvantageous to the patients; (3) measuring data for a longer period of time will inevitably bring many confounding factors, such as differences in the amount of infused fluid, the effect of individual different self-healing on the study data, the effect of different therapeutic drugs on the measured data, and so on. Third, this study has no data analysis on the prognosis of patients. The main reason is that this study is a self-controlled and observational study, consequently, the treatment plan is the same, so it is difficult to compare the difference in prognosis. There is also a lack of normal reference value ranges for PCC and PMSF, which is not conducive to the interpretation of the results of this study and remains to be further investigated to refine. Finally, analgesic used in our study contained fentanyl and sedative contained dexmedetomidine, which leads to a possibility that these medications contributed to the negative chronotropic effects, and results of this study need to be further confirmed by multicenter large sample clinical studies. Despite these limitations, this study opens a new direction for the hemodynamics study of esmolol and vascular waterfall phenomenon in the clinical treatment of sepsis patients, and the results of this study still have certain reference value in subsequent basic or clinical studies.

## Conclusions

Patients with septic shock whose heart rate remained greater than 95 bpm after hemodynamic optimization were treated with esmolol, which effectively controlled the heart rate and reduced the CI. At the same time, it can increase the Pcc, increase the difference between Pcc and Pmsf (known as “vascular waterfall” phenomenon) without affect on MAP, CVP, Pmsf as well as arteriovenous vascular resistance and it can improve the body's oxygen supply–demand balance status.

## Data Availability

The data sets supporting the results of this article are included within the article.

## Abbreviations

ABP: Arterial blood pressure
EVLWI: Extravascular lung water
GEDVI: Global End-Diastolic Volume Index
ICU: Intensive care unit
ITBVI: Intrathoracic Blood Volume Index
Lac: Blood lactate level
MAP: Mean arterial pressure
Pcc: Critical closure pressure
PICCO: Pulse indicator continuous cardiac discharge monitoring
Pmsf: Mean systemic filling pressure
RIs: Resistance Index
SI: Cardiac Stroke Volume Index
VO: Ventricular output
VR: Venous return
